# Hospital Meetings, &c.

**Published:** 1899-11-04

**Authors:** 


### HOSPITAL MEETINGS, &C.

### HOSPITAL SATURDAY FUND.

The Current Year's Finances.—The Inquiry Question.

The annual meeting of the Hospital Saturday Fund was held on Saturday at the Mansion House, by kind permission of the Lord Mayor, and was very largely attended. This being the second meeting since the discontinuance of the street collections, much interest was evinced as to the effect such abolition has had on the Fund. As was pointed out by Mr. Bonus, the chairman of the Fund, the scheme which has taken the place of the street collections is quite in its infancy, and therefore the success which has already attended it would seem to augur well for future results. Moreover, the figures given with regard to the current year, so far as it has gone, cannot but be regarded as highly gratifying, not only as to the receipts—from the point of view of their balancing the decrease occasioned by the step already referred to—but also with regard to the expenses, which are showing a sensible diminution.

Unfortunately the Lord Mayor was prevented, owing to other important engagements, from taking the chair; but those interested in the Fund received a cordial welcome from Alderman Sir Henry E. Knight, and found in him an excellent host. With him on the platform were the Ven. Archdeacon Sinclair, D.D., Sir Savile Crossley, Sir W. H. Broadbent, Mr. Bonus, Mr. George Howell, Mr. R. B. D. Acland, the Rev. C. J. Parker, Dr. Tom Taylor, Mr. J. A. Dickinson, Mr. Frank Aubrey (author of "Strange Stories of the Hospitals," presented to the fund), Colonel Clayton, Mr. C. E. Huddlestone, Mr. A. J. Symons, and several members of the council. Superintendent Rundell, Lady Superintendent Miss Mann, and the nurses of the fund division of the St. John Ambulance Brigade, also occupied seats on the platform.

In opening the proceedings, the Chairman explained the absence of the Lord Mayor, and expressed the pleasure he experienced at meeting such a large gathering of those interested in this excellent fund. He was glad to know that the contributions collected in the workshops and manufactories of this great metropolis were annually increasing in importance. That was the source from which money should come to aid this glorious institution, for they were the people who derived greater benefit than other classes from the hospitals. Another method they adopted in past years for raising funds was the public collections in the streets, and this was an excellent means when first introduced. It was, however, copied by other bodies for other purposes, and ultimately became a nuisance—(hear, hear)—and he was very glad indeed that the committee of the Hospital Saturday Fund saw their way clear to abolish the street collections, at the same time making a stronger appeal for the support of the working men of the metropolis. He was told that the annual income was something like £20,000. That was a goodly sum, but he thought he would be forgiven for saying he did not think it was anything like so much as this Fund should receive when they considered the noble purposes of the hospitals, the dangers from accident which surrounded them daily, nay, hourly, and how the working man was perhaps more subject than others to accidents in the performance of his daily duties. (Hear, hear.) He hoped the income of the Fund would largely increase year by year. (Applause.)

Mr. Bonus was called upon to make a statement of the work of the fund. He first announced the receipt of apologies for non-attendance from the Bishop of Ripon and the Hon. Sydney Holland, and one on behalf of Sir Henry Burdett, who was out of England. The income from the Fund in 1898 from all sources, he proceeded, was about £19,000, of which some £17,755 was collected in the workshops and places of business in London by means of small regular collections, in most cases amounting to 1d. per week per subscriber. The next contribution of importance was the Saturday collection, which realised £1,172. The total for the year—£19,000—was £1,000 less than in 1897, but it must be remembered that the street collection in that year brought in £3,330. In deference to public opinion they abolished the street collection, but, feeling they should not abolish Hospital Saturday, they started a new scheme which brought in the £1,172 mentioned. It must be remembered that this was a new scheme, and the receipts were not going to stop at that figure in the future. (Hear, hear.) Now, although Hospital Saturday proper showed a decrease of £2,160 for the special reason mentioned, the gross receipts showed a diminution of £1,000 only. This was owing to the general rally in the workshops, in sympathy with their action in abandoning a form of collection which had become discredited in the eyes of the public. In the end they distributed very nearly as much in 1898 to the hospitals as they had done in 1897. (Applause.) As to 1899, they had as yet no returns of the Saturday collection which would give anything like a proper appreciation of the position of that collection; but with regard to workshops, whereas in 1898 to October 22nd they had collected and paid into the Fund £11,678, up to October 21st this year they had paid in £12,461, an increase of £783. (Applause.) When they considered that their expenses for the same period were less this year by £112, they saw they were now £900 to the good as compared with last year. (Applause.) In 1898 the cost of "running" the Fund—£2,383—was less than it had been for ten years. The ratio of expenses to receipts was 12½, and although that might be regarded as a high percentage, he would point out that their work was of a completely different kind from that carried on by any other charitable institution. Of the residue they distributed £14,500 among hospitals, dispensaries, convalescent homes, and other medical charities in London; the balance was absorbed by the Distribution Committee of the Fund in purchasing letters for hospitals, by the Surgical Appliances Committee, and by the Ambulance Committee. As to their relations with the outside world, the public, for whose convenience they abolished the street collections, had made no particular return to them for giving up the £3,330. The substituted system of Saturday collection consisted largely in placing boxes in banks, shops, &c., for three weeks in October; but these boxes did not bring in the return that might reasonably be expected. One box brought in the handsome sum of 1½d. Their relations with the other collecting funds had during the past year been absolutely cordial, and with regard to the relations with the hospitals, there was a resolution on the agenda referring to inquiries made by patients, which showed that the Fund was not obstructive in the matter. The resolution was, in substance, identical with that passed several months ago by the Board of Delegates of the Fund, and it was brought forward that afternoon with a view to endorsing the decision then arrived at. (Applause.) They were doing their best to solve the great out-patient question. As an instance, they had adopted a system by which applicants for chest hospital letters were in the first place sent to provident dispensaries. The difficulty as to out-patients was twofold. First, people came who were not financially in a position to apply for gratuitous aid, and against that difficulty the inquiry system was levelled; secondly, people came who were not medically the right people to be treated at hospitals. By distributing applicants among dispensaries they might get them just as good treatment as they would obtain at hospitals, and at the same time they would be greatly relieving the hospitals.

The Venerable Archdeacon Sinclair proposed the following resolution: "That this meeting, recognising the interest which the workers of the metropolis are taking in the medical charities of London, confidently appeals to the working-classes through the Hospital Saturday Fund for continued and increased support, in order to obviate any loss to the Fund by the discontinuance of the ladies' street collection, and to enable the fund to still further extend its beneficial work." He characterised the fund as a useful and admirable institution, and spoke of the importance of the working men of London realising their duty and privilege with regard to it. He declared that the attitude of the Christian Church was one of whole-hearted sympathy for any great work of charity such as this, and urged upon his auditors the great and increasing need for the support of the hospitals.

Sir Savile Crossley, one of the secretaries of the Prince of Wales's Fund, spoke of the cordial relations which existed between the two institutions, and of the great advantages of these central funds. He alluded to the satisfactory progress made by the Prince of Wales's Fund and to its improved prospects, and congratulated those present on the excellent work done by the Saturday Fund. He explained the position of his own fund with regard to the question of inquiries of patients at hospitals, pointing out that while actual inquiry on their part was quite out of the question, they did their best to ascertain what steps were taken by the hospital authorities themselves in the way of appointing an almoner or an inquiry officer. He cordially approved of the abolition of the street collection, and trusted that that action would not be a source of loss but rather one of very great gain to the Fund. (Applause.)

Mr. George Howell supported the resolution, remarking that if each one did his duty the call would fall lightly upon all.

The resolution was carried unanimously.

Mr. R. B. D. Acland moved: "That this meeting of the supporters of the Hospital Saturday Fund is not adverse to inquiries being made into the fitness of all patients applying for medical relief, provided that the medical condition of the patient is regarded as of primary importance and that any further investigation is made with tact and due consideration for the feelings of the patients and their families." There had, he said, been so many misrepresentations as to the attitude of the Fund with regard to this question that the Council had thought it desirable to submit such a resolution. The delegates and subscribers to the Fund were as anxious as the sturdiest and most hard-working hospital secretary that no person should be sent to the hospitals who was not a proper object of hospital treatment. They were not engaged, and did not desire to be engaged, in a battle with the hospitals to force upon them persons who were not fitted, either for medical or social reasons, to become the recipients of the benefits of these institutions. They only desired that if inquiries were made the two conditions mentioned in the resolution should be regarded.

Sir William Broadbent seconded the resolution, which, however, he did not consider strong enough. He thought they might have gone further, and said they gave the inquiry system their cordial support.

Mr. Manfred Denvil, president of the Cobden Working Men's Club, Kensal Road, W., spoke to the resolution, which was carried unanimously.

The Chairman then presented diplomas of merit to Mr. James Thorley and Mr. William Rumsey for conspicuous services in ambulance work. He remarked that such a diploma was a thing any man should be proud of; he would be only too glad if he had the chance of getting one himself. (Laughter and applause.)

A cordial vote of thanks to the Lord Mayor for the use of the hall, and to the worthy Alderman for presiding, closed the proceedings.

### EVELINA HOSPITAL FOR SICK CHILDREN.

A meeting of the governors was held on Wednesday evening, the 25th inst., at the hospital, Southwark Bridge Road, S.E. Mr. F. W. Reynolds, J.P., presided in the absence of the Hon. Conrad Dillon (chairman Committee of Management).

The Chairman said that at the present time they had £516 10s. 6d. in hand, which shortly, in consequence of certain payments, would be reduced to £23 18s. 11d., and £1,000 on deposit. Therefore the hospital was urgently in need of subscriptions. Since the announcement of the splendid legacy of £100,000 by the late Baron Ferdinand Rothschild, donations had somewhat fallen off, and the interest on the £100,000 would not nearly compensate them for the pecuniary assistance rendered by the late Baron to the institution. There were 65 children at present in the hospital, and 1,655 out-patients had been treated during the past fortnight. The usual number of in-patients years ago was about 50. It was necessary to elect a trustee in the place of their late president and founder, and Mr. Leopold Rothschild had advocated the appointment of Mr. Charles Cotes, a member of the Stock Exchange, and a gentleman who had interested himself in hospital work. In conclusion he moved, "That Mr. Charles Cotes be elected a trustee."

Mr. J. Arthur Dawes seconded the motion, which was unanimously agreed to.

A vote of thanks to the chairman closed the proceedings.

